# Motor control and skill acquisition in pediatric physical therapy: an enactive proposal

**DOI:** 10.3389/fpsyg.2023.1226593

**Published:** 2023-10-13

**Authors:** Marit Sørvoll, Gunn Kristin Øberg, Gay L. Girolami

**Affiliations:** ^1^Faculty of Health Sciences, Department of Health and Care Sciences, UiT The Arctic University of Norway, Tromsø, Norway; ^2^Department of Clinical Therapeutic Services, University Hospital North Norway, Tromsø, Norway; ^3^Department of Physical Therapy, College of Applied Health Sciences, University of Illinois at Chicago, Chicago, IL, United States

**Keywords:** motor control, skill acquisition, embodiment, participatory sense-making, pediatric physical therapy

## Abstract

Theories of motor control and skill acquisition strongly influence and guide various fields of clinical practice. In last decades, changes in theoretical frameworks related to the conceptualization of brain plasticity, functional structures within the child, and environment have led to a revision of therapy approaches progressing from therapist-driven to child-initiated approaches. Even though theoretical frameworks and clinical practice are closely linked to the child’s body, the profession has paid less attention to theories concerning the body’s role and status in interpersonal relationships when fostering motor control and skill acquisition in children. In this theoretical paper we discuss the theoretical frameworks of motor control and skill acquisition that currently guide clinical practice. Through highlighting valuable contributions of these theories, we explore theoretical and practical benefits pediatric physical therapy can acquire by taking an enactive approach as a means to bring the child as a subject into focus. We rely on enactive concepts of embodiment, autonomy, and participatory sense-making in our exploration to provide an extended understanding of motor control and skill acquisition shaping our beliefs about what counts in therapeutic encounters in pediatric physical therapy.

## 1. Introduction

Worldwide in pediatric physical therapy we have many theoretical frameworks to guide our thinking about ways to enhance motor control to achieve optimal development and skill acquisition. The availability of numerous theoretical frameworks has had implications for how we approach and plan interventions for various pediatric conditions (Palisano et al., 2023). Over the years these theories have changed, and implicit in these changes are the conceptualizations of the role of brain plasticity and functional structures within the child. Also significant in recent theories is discussions regarding the importance of the environment and task selection on the developmental trajectory, which generated discussions on the merit of therapist-driven versus child-initiated approaches (Palisano et al., 2023).

Currently the three primary theoretical frameworks discussed in physical therapy related literature are dynamic systems-, perception-action-, and neuronal group selection. These three theories incorporate the most up to date research about motor control and development, aggregated under the umbrella of dynamic systems approaches (Stergiou et al., 2006; Palisano et al., 2023). Dynamic system approaches have contributed important knowledge to our understanding of development of motor control as interactional self-organizing processes among many autonomous systems, at many levels, on different timescales to improve function and active participation of the child (Thelen, 1995; Palisano et al., 2023). However, within dynamic system approaches the development of motor control and skill acquisition in children has been viewed from a physical perspective of the body (e.g., length and strength) and an objective assessment of movements (e.g., amplitude, duration, and speed) while the children’s subjective lived experiences and sense-making are not addressed. For example, in the definition of the concept of self-organization, the individual or the self is absent, i.e., contributions from the subject are not considered as part of the self-organizing process (Thelen, 1995; Thompson, 2010). Therefore, an important aspect of the body is not addressed, i.e., how the body pre-reflectively (without conscious reflection) feels, senses, and perceives itself subjectively as a basis for regulating itself in relation to others and to the environment (Fuchs, 2020). This raises questions regarding how to improve children’s pre-reflective awareness of their bodies, during interactions with others and the environment, to develop, perform, and master tasks in a meaningful way? How can the child’s body be the means through which the child can inhabit the world? What implications will the understanding of a bodily subject for motor control and skill acquisition have for the inclusion of intersubjectivity and inter-corporeal relationships between the child and the physical therapist (PT)? The aim of this paper is to describe and discuss an enactive theoretical framework, which brings the child as a subject into focus, as a means answering the above questions (Fuchs and De Jaegher, 2009; Di Paolo et al., 2010, 2017).

To enact is how human beings bring forth a world of significances through (inter)actions rather than being receivers of inputs from the environment (Varela et al., 1991). By taking an enactive view, we can form a more comprehensive understanding of motor control and development as subjective and intersubjective circular processes arising through movement, perception and (inter)action. Although contemporary descriptions of motor control and skill acquisition in children may use terms that correspond to terms in the enactive approach, e.g., embodiment, embedded, and agency (Thelen, 1995; Adolph and Hoch, 2019; Palisano et al., 2023), we find that the conception of these terms is too narrow as they merely refer to the objective physical body and do not fully explain the important concepts conveyed by the enactive approach, e.g., autonomy (inter)subjectivity, incorporeity, and participatory sense-making (Fuchs and De Jaegher, 2009). Through the lens of the enactive approach, these aspects are important as they allow us to fully comprehend and reconceptualize our understanding of the child as an action-oriented embodied subject in the world, and therefore expand our understanding of motor control and development. Furthermore, the enactive perspective resonates with the complex features of clinical practice in pediatric physical therapy and will change how we look at the application of clinical practice.

In the first section of this theoretical paper, we provide a brief overview of the dynamic systems approaches and where these theories fall short in addressing the self in development of motor control and skill acquisition. In the second section of the paper, we present the enactive approach by discussing concepts of embodiment, autonomy, and participatory sense-making to expand our understanding of the importance of considering the role of self in development of motor control. In the last section, we discuss the application of an enactive approach to the practice of pediatric physical therapy using hypothetical examples to illustrate the significance of recognizing and acknowledging the child as an embodied subject. We acknowledge that there are several genders but to make the text easy to read we will refer to the child as a she/her/herself throughout the article.

### 1.1. Current understanding of motor control and skill acquisition

The construct of motor control and skill acquisition is a crosspollination of a broad range of disciplines including anatomy, physiology, psychology, and physical therapy (Palisano et al., 2023). Motor control and acquisition of skills include the development of postural control and the production of useful coordinated movements needed to foster participation in daily life (Palisano et al., 2023). Each of the dynamic system approaches describes the foundation for motor control and skill acquisition, however, despite several common features (e.g., the child in relation to the environment and self-organizing systems within the child), each theory has its own explanation of how motor control and skill acquisition evolve.

Dynamic systems theory emphasizes process rather than the product with no one system (e.g., musculoskeletal system, sensory system, etc.) considered superior to another in fostering motor development (Thelen, 1995; Palisano et al., 2023). In short, motor development is driven by the unplanned discovery of movement possibilities and the flexibility to select the most optimal movement synergies to achieve a desired outcome (Palisano et al., 2023). In this context, the concept of self-organization based on patterns of motor behavior is not considered pregiven but emerging through the intersection of the task specifics, the context/environment, and the abilities of the individual/organism (Thelen, 1995; Shumway-Cook et al., 2023). The environment is as important as the individual/organism. In this manner multiple pathways can lead to developmental changes, such as biological predispositions, environmental causes, motivational states, adaptation, and survival (Narvaez et al., 2022; Palisano et al., 2023). Through repetition and practice, interacting systems organize themselves and create movement repertoires. Development of motor control and skill acquisition is therefore understood as fluid and variable, with no preferred motor solution applied within or across different circumstances (Thelen, 1995; Darrah and Kembhavi, 2022; Palisano et al., 2023). The self-organizing powers driving the development of a child’s behavior are viewed as processes that are automatically organized without conscious input from the child (Smith and Thelen, 2003). Thus, there is an agent-environment system, but the action-oriented subject/the self is missing within this integrated system. The child as a sensemaking subject is not incorporated in this theory.

In the neuronal group selection theory, the brain is considered important for development (Hadders-Algra, 2000; Palisano et al., 2023). This theory acknowledges the interaction of factors represented by the child, task, and environment as contributors to functional changes in the brain, which support motor behavior (Hadders-Algra, 2000, 2018). In this sense behavior is considered unique to the individual because of variation in the neuronal maps shaped by individual experiences in response to environmental demands and physiological changes in the child (e.g., growth). Thus, the neuronal group selection theory emphasizes the concept of selection; the brain can select behaviors that are useful and have value to the individual based on movement experiences, i.e., when the individuals interface with their environment the brain creates neuronal maps to support the effective movement strategies (Hadders-Algra, 2000, 2018). Stereotypical movement patterns provide poorly differentiated brain maps while varied movement patterns lead to rich and complex brain organization (Hadders-Algra, 2000, 2018). In sum, the neuronal group selection theory highlights how the anatomical structure and organization of the brain are affected by experience and environmental demands and does not incorporate the child as a bodily sense-making subject.

The third dynamic system approach, perception-action, describes perception and action are mutually and reciprocally related, i.e., what the child sees and perceives, shapes how she organizes movement to approach or acquire a specific object or person (Adolph and Hoch, 2019; Palisano et al., 2023). This in turn shapes her perception of what she can do and through repetition and practice supports successful movement organization and eventually the development of motor control and skill acquisition (Adolph and Hoch, 2019; Darrah and Kembhavi, 2022; Palisano et al., 2023). In this theory, perception is based on the processing of sensory stimuli, e.g., sensation of movements, sensation of color, sensation of size, etc., providing the perceiver “indirect” access to others and the world through representations or inferences (Fuchs and De Jaegher, 2009; Gallagher, 2017). That is, the representations produced by the various sensory inputs first converge and then are modulated by perception before the unified result proceeds to cognition. Based on current and previously stored input a motor plan is created and then executed as an action (Hurley, 2001). Thus, this theoretical approach highlights specifically generated perception-action loops in a representational way based on sensory inputs followed by actions. These loops involve the individuals’ body as the means of developing motor control and skill acquisition through cyclical movement patterns, which build skills through repeated, self-generated movements and the child’s understanding of affordances in the environment. However, these perception-action loops do not account for an understanding of perception as an immediate and pre-reflective (not conscious reflection) bodily way to understand others and the environment and do not encompass various forms of sense-making related to non-representational dynamical processes, affect, embodiment, and intersubjective relationships (Adolph and Kretch, 2015; Di Paolo et al., 2017).

**Figure 1:**
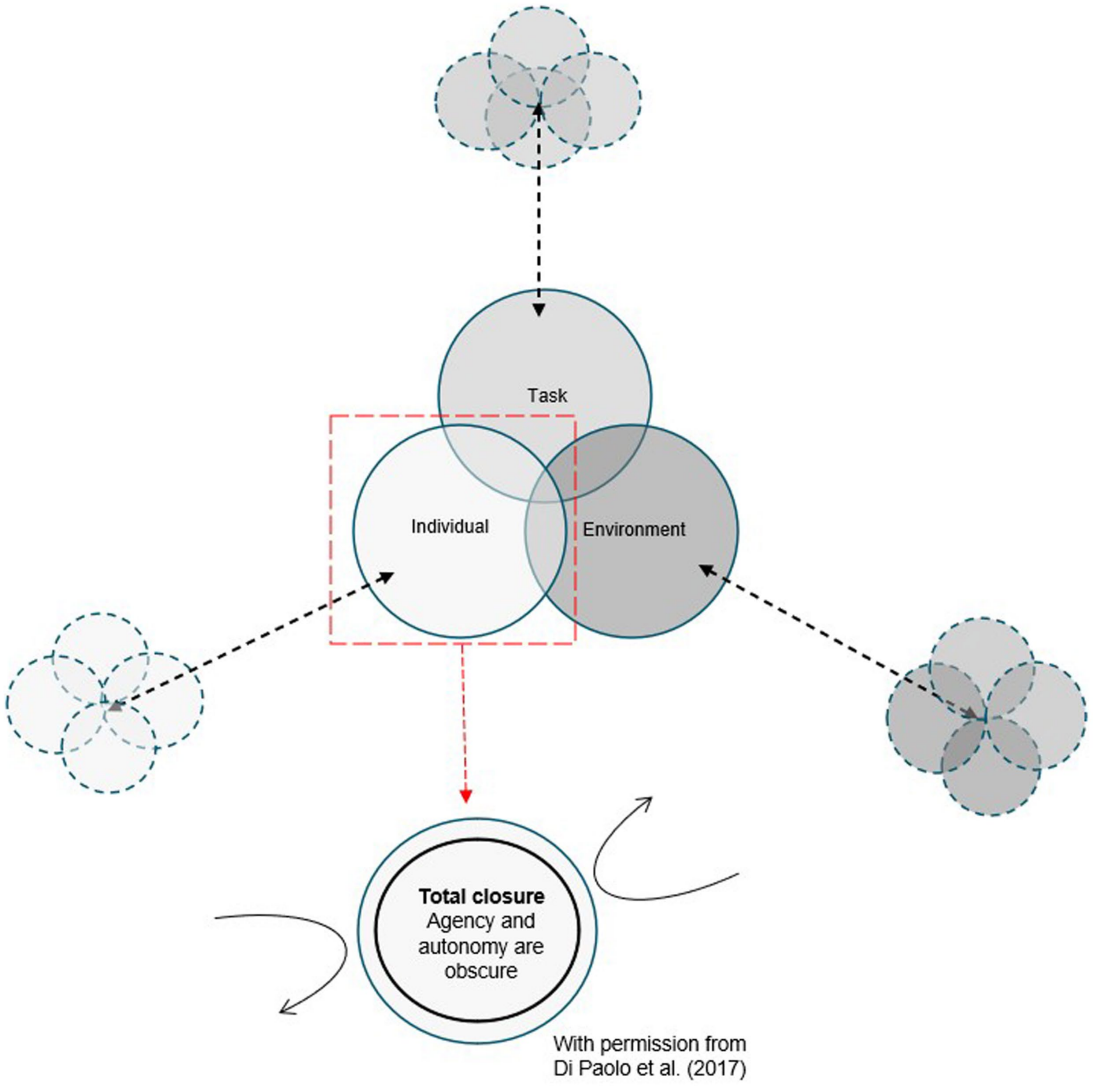
This figure is inspired by and pieced together by illustrations of dynamic systems commonly used by PTs worldwide (Shumway-Cook et al., 2023) and by the work of Di Paolo et al. (2017). The large illustration is an example of how the PT literature commonly visualize the emergence of movements (and thus sensorimotor development) from the interaction of factors divided into three overlapping systems (i.e., three large circles): an individual system generating movements from task and environmental demands. Each system is further connected (via a black dotted bidirectional arrow) to an architecture of new overlapping sub-systems (i.e., four small overlapping circles) considering important factors for the organization and function for each individual system. The sub-systems are typically not interconnected to the other domains of sub-systems. In the context of physical therapy, the individual refers to the patient (e.g., the child) and appears (from the illustration) to be a closed pregiven and fully developed system overlapping but not interacting and co-regulating with the task and the environment. Thus, the individual, the task, and the environment appear as three distinct independent systems. As further visualized and demonstrated by the illustration at the bottom (2017), the individual strongly self-distinguishes and, as such, is an impenetrable system. Accordingly, this prevents any task-environmental flow (represented by black curved arrows) affecting the system and vice versa. How the system self-produces are therefore unclear, making the autonomy, sense-making, and the agency of the system obscure. Additionally, a visual other (e.g., the PT) is missing providing an impression of a non-social environment.

Common and inherent in these three theories, is an understanding that development is driven by a connection between different systems, i.e., the maturation of the psychological and physiological systems within the body and the demands placed on children by the environment and task-related experiences (Darrah and Kembhavi, 2022; Palisano et al., 2023). Each of these three theories are based on a three system model of task, individual, and environment, which is commonly highlighted in the pediatric physical therapy literature (see Figure 1) (Shumway-Cook et al., 2023). However, the role of the child and what motivates her and what she feels and experiences are not given attention. There also seems to be a stronger emphasis on the task and the environment driving the child versus the child using her experiences in a way that makes her aware of what her body can do and what she wants to do and how she needs to move to do and accomplish it. Consequently, this has led to a hypothesis that the child’s sensorimotor development evolves by itself if the PT facilitates the environment and the task (VanSant, 1996). This hypothesis seems to have contributed to an understanding of the PT as a facilitator (arranging environment and tasks) rather than being actively involved with the child throughout the intervention process (Akhbari Ziegler et al., 2019), thus minimizing the importance of touch in physical therapy practice. However, this is in opposition of the view of physical therapy as deeply grounded in a culture of touch to make a connection with the patient and facilitating and guiding movement (Sørvoll et al., 2022). The shift towards emphasizing facilitation of environment and task as a vital component of clinical practice has contributed to a move toward the use of hands-off versus hands-on interventions and task-oriented approaches in pediatric physical therapy.

Touch involves specific therapeutic hands-on techniques to enable and foster movements. For example, touch to increase sensory stimulation, for tissue mobilization, and to address and support sensorimotor systems, particularly in the child with neuromotor and developmental delays (Nicholls and Holmes, 2012). As an added benefit, touch can foster attention, interest, and engagement (Øberg et al., 2013; Bjorbækmo and Mengshoel, 2016; Sørvoll et al., 2022). Defined in this way, touch becomes a form of mutual communication between the PT and the child where the PT adapts the pressure, velocity, and direction of the touch to the child’s bodily needs, expressions, and responses to support and guide the child’s body and movements (Sørvoll et al., 2022). However, recent discussions in the physical therapy literature argue that touch as a therapeutic tool may make the child an inactive recipient of treatment decreasing engagement and skill acquisition, thereby, providing the impression that touch should be avoided (Longo et al., 2019; Novak et al., 2020). Thus, although dynamic systems approaches contribute important aspects regarding the development of motor control in children, the child as a socially participating bodily subject is not recognized in these theoretical frameworks, thereby diminishing the awareness of the child and touch as a way to communicate.

### 1.2. Cartesian knowing and the outside observer

The theoretical basis of motor control and skill acquisition in dynamic system approaches rests mainly on ontological (what exists) and epistemological (ways of knowing) assumptions inherited from a Cartesian dualism (Varela et al., 1991; Thompson, 2010; Nicholls, 2017). The Cartesian dualism describes mind and matter/body as independent substances (Varela et al., 1991; Thompson, 2010). In this view, the center of knowing resides in the mind (Thornquist and Kirkengen, 2015). Related to physical therapy, the current theoretical underpinning of physical therapy education is largely organized around a biomechanical view of the body, i.e., the body-as-a-machine, detached from cultural, social, and contextual surroundings (Nicholls, 2017; Maric and Nicholls, 2020; Richter and Maric, 2022). From the Cartesian view the main property of matter/body is its spatial extension while the property of mind is its capacity to think. This means that knowledge of reality (cognition) is connected to a mind that resides in the brain (Varela et al., 1991). Thus, the distinction supports a view of nature (e.g., human bodies, plants, minerals, etc.) from a third-person perspective (the observer perspective) in which nature is viewed as a mechanism composed of independent parts, for example, the child as matter/an objective body, i.e., merely a collection of separated layers, entities, and functions like cells, organs, muscles, limbs, etc. Richter and Maric (2022). Such an epistemological split between a subjective and objective reality, between the world within and the world outside, separates the knower, i.e., the subject and her interior awareness, from the known, i.e., the exterior reality/the object of knowledge (Varela et al., 1991). In physical therapy then, the biomechanical view of the body provides the conceptual ground for physical therapy practice justifying the PT’s role as an expert who knows how to solve the patient’s problem (Nicholls et al., 2023). From a PT perspective, knowledge about the body as matter (e.g., muscles, limbs, and neuro-muscular function) is essential for observing the child’s movements in different postures, and completing various tasks. The PT (in this example, the knower) gathers information and forms hypotheses and strategies by observing and assessing the child. However, this third-person perspective of the child is insufficient as the child as a subject can be ignored. Hence, the child’s body (the object of knowledge) is viewed mechanically (i.e., body-as-a-machine). Therefore the PT hypothesizes the therapeutic needs of the child by addressing independent parts of the child’s body and incorporating the PT’s idea of the child’s inner state to achieve what the PT believes is a holistic understanding of the child. Consequently, the child becomes an idea within the PT’s consciousness, i.e., an inner representation or mental construct that resides in the PT’s brain (Brender, 2013). In line with the cartesian assumptions, the PT’s brain becomes superior, the residence for the mind/consciousness and thereby cognition, which in turn leads to a PT-child relationship in which the child as a bodily subject, i.e., an experiencing, expressing, and communicating mind–body, is disregarded.

By simplifying the role of the individual (child) to an integrated individual-environment system (see Figure 1), which aligns with the conceptualization of the dynamic system approaches in pediatric physical therapy, the role of the therapist has become blurred. Is the PT part of the task or part of the environment? Clarifying the PT’s role is important because it affects the way therapeutic interventions are planned and executed. As stated earlier, the cartesian underpinning of dynamic system approaches have contributed to the currently expressed belief that the PT should take a hands-off, observational role of facilitating the child’s self-organizing development through the arrangement of tasks and enriched environments to engage the child in her own development (Akhbari Ziegler et al., 2019; Novak et al., 2020). However, this may work for some children, but it will not work for all children. For example, children with neurological motor impairments, e.g., children with cerebral palsy (CP), the ability to actively engage with and influence their surroundings to shape their own experiences and learning, can be challenging due to reduced postural control, which inhibits the production of useful, variable, coordinated movements and the acquisition of skills (Palisano et al., 2023). Further, the cartesian underpinning of dynamic system approaches may also have led to assumptions about clinical practice whereby PTs justify their working knowledge solely based on theories with predominance of a biomechanical (cartesian) third-person facts, potentially ignoring other important aspects of how to foster motor control and skill acquisition in children. To emphasize and meet the child as an expressing, experiencing, and sense-making bodily subject involves co-creation of meaning through interaction processes and various modalities of touch (Sørvoll et al., 2022). In this scenario touch is not solely about a physical touch itself, but becomes a way to communicate through gaze, bodily expressions, handling (pressure, velocity, and direction of the physical touch) and facilitation of the task and environment adapted to the child’s initiatives, bodily expressions, and bodily responses (Sørvoll et al., 2022). We therefore argue that involving the child as a social, autonomous, and experiencing agent will enhance and expand the understanding of how PTs can foster motor control and skill acquisition in children. In addition, this view of the child also influences how PTs support and acknowledge the child’s embodied self and thereby optimizing the child’s motor performance. We believe that embracing an enactive reconceptualization of the body, which views the mind/consciousness as distributed across the brain–body-environment (Di Paolo et al., 2017) is required to embrace the child as an autonomous agent interacting with her environment in a reciprocal manner.

### 1.3. Bringing the body into the center

Through the lens of the enactive approach and its phenomenological branch, embodiment implies that the child always is in the world as a mind–body, dynamically interacting with the environment. The locus of cognition is expanded from residing in the brain to a ‘mind–body-environment’ synthesis (Fuchs and De Jaegher, 2009). Thus, embodiment includes a wide range of bodily processes (including sensorimotor- and affective ones) in cognition as well as in the mind–body dynamic relationship with others and the environment (Gallagher, 2017). That is, the mind can be both a living and a lived phenomenon that evolves from the coupling between an agent/child and her world (Thompson, 2010). The child as a living body comprises observable, biological dimensions, while the child as a lived body involves her first-person phenomenological or experiential properties (Fuchs, 2020). Therefore, the living body and lived body will always be mutually related and intertwined, and so too, the cognitive system resides in both the living body and lived body forming an integrated and complex whole (Fuchs, 2020).

**Figure 2:**
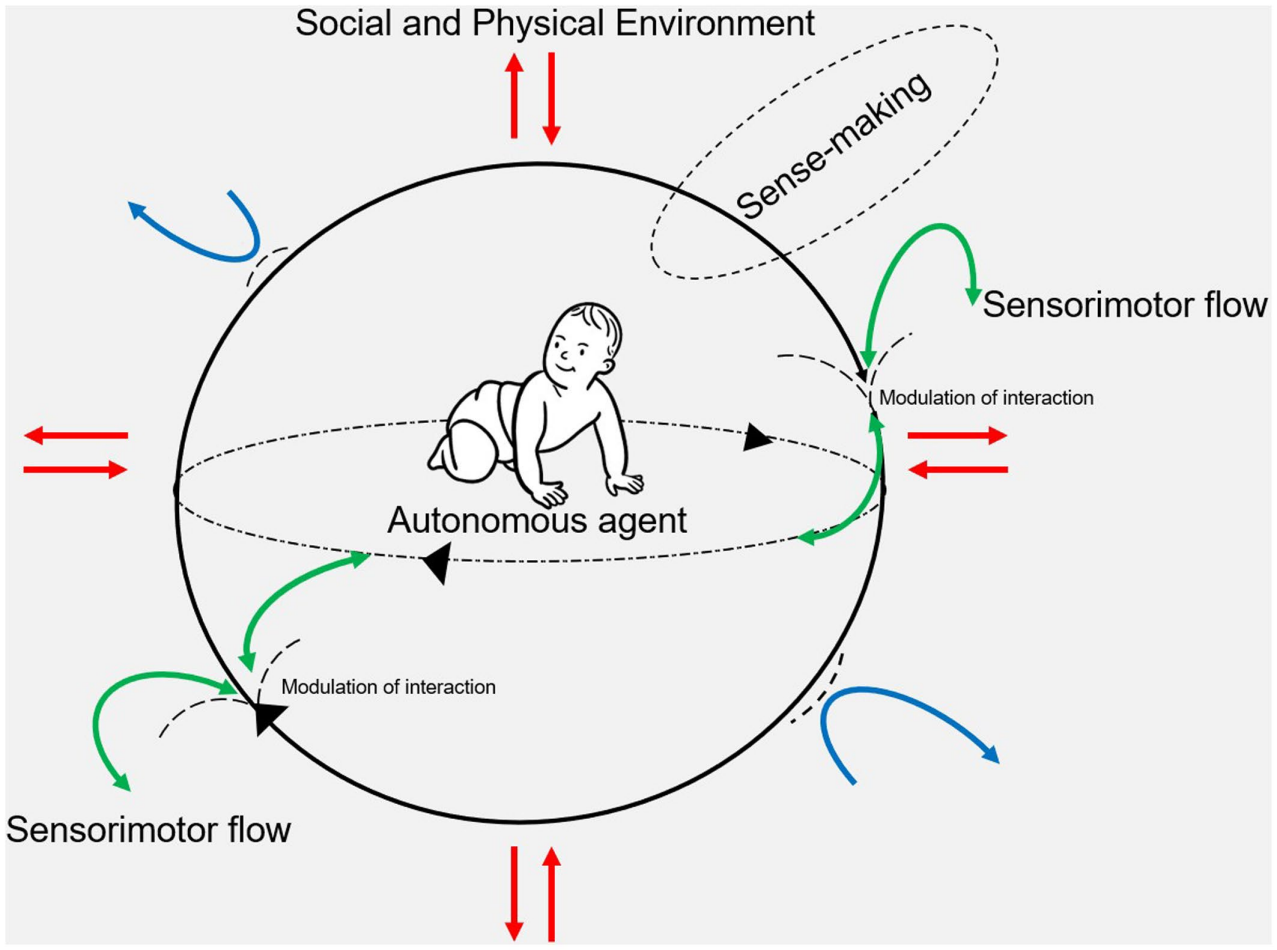
This figure is inspired by the work of Di Paolo et al. (2017). The social and physical environment are represented by a grey background. The two embedded circles illustrate an embodied, autonomous, and adaptive agent (e.g., child) producing and maintaining herself by regulating her metabolic exchanges and sensorimotor interactions with the social and physical environment. The two-way coupling between agent (e.g., child) and environment is represented by the red arrows. Adaptive regulations with the environment enable the autonomous agent (e.g., child) to be open to selected environmental flows (contributing to self-production) and closed to others (contributing to self-distinction). Rejected environmental flow is represented by the blue curved arrows, while the green curved arrows represent accepted environmental flow visualized as the modulation of sensorimotor in- and outflow. Through the agent’s (e.g., child’s) re-entrant activity, she defines her own enabling conditions fostering and sustaining her identity according to vital norms and goals and thus constitutes herself as a sense-maker. Sense-making is an ongoing activity of creating value and significance and co-emerges with the agent’s (e.g., child’s) engagement and activities.

Taking an enactive approach, accepting there is a *living* and a *lived* body implies acceptance of an extensive and alternative understanding of motor control and skill acquisition that recognizes mind/consciousness as constituted by embodied movements and actions (Gallagher, 2005). Bodily movements and actions thus become a path of communication and bonding (e.g., between the child and the PT), justifying and legitimizing the inter-subjectivity of interpersonal relationships as part of the understanding of motor control and skill acquisition in daily life as well as during physical therapy. Further, as part of bodily becoming, circular networks of relations and flows within and between the system and its coupling to the environment are conceptualized as circularity by the enactive approach (Di Paolo et al., 2017; Fuchs, 2020) (see Figure 2).

The enactive concept of circularity highlights how various structures and dynamics of the organism, such as regulatory cycles between the brain and body at multiple levels, intwine the lived body with the living body and thus foster an embodied sense of self (Fuchs, 2020). There is a continuous mutual resonance between the brain and the body, i.e., various areas of the brain (e.g., the brain stem, the hypothalamus, and the insular cortex) process afferent information from the body (e.g., proprioceptive, visceral, and endocrine information) forming a background consciousness/state of the body (e.g., muscle tension, vestibular sensations, temperature, and heart rate) (Fuchs, 2020). It is important to note that this background consciousness is not a mental image or internal model of the body located within the brain, but an integral manifestation or resonance of the brain–body system (Fuchs and De Jaegher, 2009; Fuchs, 2020). Conversely, the brain regulates the homeostasis of the body through the parasympathetic and sympathetic nervous system and hormone secretions. This vital self-regulation of the body (circular feedback-loop) demonstrates the close interaction between the brain and the body and how this interactive loop gives rise to a minimal form of subjectivity (Fuchs, 2020). Therefore, in the enactive concept of circularity, the subjective lived body arises from the interactive loop of the living physical body through self-regulatory cycles, highlighting the animate aspect of the body; how the body pre-reflectively self-sustains, feels, senses and perceives itself as a basis for regulating itself to the environment (Fuchs, 2020).

The enactive concept of circularity also highlights cycles of sensorimotor couplings between the child (organism/body) and her environment (Fuchs, 2020). This implies a view of embodied subjectivity that goes beyond the boundaries of the body intwining with the environment (Merleau-Ponty, 1962). In that context, the lived body is pre-reflectively experienced as the center of sensorimotor perception and (inter)action, in which the bodily sense of self becomes an extended pre-reflective consciousness directed towards the world (Fuchs, 2020). According to the enactive approach, cognition, perception and bodily movements constitute processes of sensemaking (Fuchs and De Jaegher, 2009). From this viewpoint sense-making is considered an agent’s perspective of meaning on a world invested with interest, value and significance all generated through the agent’s body and actions (Di Paolo et al., 2017). By enacting the world (i.e., creating significance through our interactions with the world), sense-making processes are constituted by a simultaneous awareness of bodily movements and perception; the brain–body-environment system is operating within the situation itself rather than on a mental representation of the situation inferred by the brain (Gallagher, 2017). This means that perception is always direct and immediate through bodily movements, and that the environment represents a dynamic structure of appearance. In this way, what unfolds in the moment can have a different value for different children based on their sense-making processes. However, the child’s perceptions of her surroundings will also correspond to the capacities of her body. That is, a toy can only be perceived as useful or a possibility if the child can interact with it, that is, having the motor control needed to reach for and grasp it, and so forth.

In sum, an important part of human life is characterized by being a body that is material, dynamic, and self-organizing, while being simultaneously animated and affected by itself and the world (Di Paolo et al., 2017). As such, the body is constituted by the experiences, socio-linguistic practices, and socio-environmental relations, and is thus, an on-going and becoming structure of situated powers and sensitivities (Di Paolo, 2021). Human beings thereby develop their own structural embodied features that enable specific and individualized perception-action loops with social and physical environments (Gallagher, 2017). The child as an experiencing, expressing, and intentional body is therefore always situated and directed towards something or somebody in the environment, indicating that sense-making is not a pure conscious process but an intrinsic aspect of lived bodily movements and (inter)actions (Gallagher, 2012), also conceptualized as ‘sensorimotor life’ (Di Paolo et al., 2017).

## 2. An enactive approach to motor control

Sensorimotor life then, is defined as the life the child lives while she is engaged in performing movements, exploring the environment, and interacting with others (Di Paolo et al., 2017). The child is exploratory by nature and has the curiosity to explore through all her senses (touch, smell, vision, etc.) (Sørvoll et al., 2022). As the child regards objects, herself, and others through perceptual bodily movements, motor control is at the core of the (inter)actions and her sense-making and applies in every situation the child encounters. Sensorimotor processes allow the child to form and sustain intersubjective interactions (Di Paolo et al., 2017). Therefore, the child is able to be reciprocally directed toward other individuals and the environment when perceptual bodily movements are supported by adequate motor control. Initially, the young child does not execute actions to achieve a clear goal but experiences the world through trial and error (Palisano et al., 2023; Shumway-Cook et al., 2023), facilitating her own actions as an adaptive, autonomous, and self-generating agent. To gain a greater understanding of the lived body as it relates to the development of motor control, we will present enactive concepts of autonomy, lived body, and participatory sense-making, which we consider are critically important for the practice of therapy.

### 2.1. Autonomy, lived body, and participatory sense-making in motor control

In the formation of bodily becoming, the body as an autonomous system (organism) self-organizes and self-produces itself by regulating its metabolic exchanges and sensorimotor interactions with the social and physical environment (De Jaegher and Di Paolo, 2007; Di Paolo et al., 2017; Fuchs, 2020). Thus, autonomy is about vulnerability and fragility in the process of self-sustaining and is fundamental for human existence because human beings are intrinsically vulnerable to change and to being changed (Di Paolo et al., 2017; Beer and Di Paolo, 2023). This makes human beings vulnerable and fragile in their engagements and interactions with others. In and through (inter)acting with others, the child as an autonomous lived body understands herself and others because she is emerging, changing, and growing during these interactions.

The interaction process, conceptualized as participatory sense-making, plays a central role for the generation of meaning and understanding (Fuchs and De Jaegher, 2009). Thus, the concept of participatory sense-making is defined as intersubjective embodied processes whereby the sense-making happens through processes of sensorimotor coordination and movements by two or more individuals (De Jaegher and Di Paolo, 2007; Fuchs and De Jaegher, 2009). This sensorimotor coordination involves various degrees of mutuality in participation. On the one hand, in unilateral coordination the individual’s attention is ‘coordinated-to’ another person, event, process, etc. with a low degree of mutuality. On the other hand, co-regulated coordination (‘coordination-with’) involves coordinated joint attention and joint action processes between the child and her peers, caregivers, the PT, or others with a high degree of mutuality. This spectrum of mutuality has significance for the individual’s sense-making processes and the interaction process itself gains its own autonomy (De Jaegher and Di Paolo, 2007). Sometimes infants participate in coordinated interactions where caregivers guide them, while in other cases, the infants proactively lead and/or influence the interaction. The degree of participation is important for the modulation of individual sense-making (De Jaegher and Di Paolo, 2007; Fuchs and De Jaegher, 2009). The child and the PT as intentional and embodied individuals understand and make sense through the coordination and synchronization of movements in the face-to-face interaction. As such, movements, posture, gestures, affect attunement, facial and vocal expressions enable the individuals (e.g., child and PT) to express intentionality (Fuchs and De Jaegher, 2009). Accordingly, the understanding of the autonomous developing lived body needs to be developed in more details to highlight the connection between the lived body and participatory sense-making.

From the beginning of life, the autonomous child communicates through her lived body via postures, movements, and touch, fostering important relationships for participatory sense-making (Quintero and De Jaegher, 2020; Sørvoll et al., 2022). For the child to be oriented towards, participate in, and interact with her social and physical environment, her lived body is dependent on and linked to sensorimotor coordination, touch and movements (Merleau-Ponty, 1964). The emerging capacities of sensorimotor coordination and movements are related to two interacting and developing systems, body schema and body image, which structure a child’s consciousness, i.e., her proprioceptive awareness of herself. These interactive developing systems, body schema and body image (Gallagher, 2005), contribute to the child’s autonomous being in the world. Body schema is a non-conscious system of sensorimotor capacities that enable the child to move and explore her surroundings while body image is, in the early stage of life, a proprioceptive awareness and perceptual experience of the child’s own body and gradually develops into perceptions, attitudes, and beliefs about her own body (Gallagher, 2005). A well-functioning and emerging body schema contributes to the child’s posture, balance, and movements and is continuously evolving and adapting to the interactions between the embodied child, others, and her environment. Body schema develops outside of the conscious awareness of the child and allows the child to be more directed toward her surroundings than to her body. Initiation and modification of the body schemas are based on continuous processing of sensory information through touch, perception, and movements (Gallagher, 2005). Therefore, the child’s sensorimotor repertoire and body schemas evolve as increasingly organized sets of coordination patterns and emerge, shape, and adapt through the timing, speed, duration, etc. of the child’s experiences. Body schema and the body image, continuously shaped by movement experiences, are central to the development of intersubjectivity (Gallagher, 2005) and thus participatory sense-making. The process of intersubjectivity includes the generation of meaning through a mutual incorporation of the individual’s lived body, i.e., the lived body transcends itself and partly intwines with the other individual’s lived body (Fuchs and De Jaegher, 2009).

Sensorimotor capacities for perception and behavior are already shaped at the time of birth as a result of the infant’s prenatal movements (Quintero and De Jaegher, 2020; Sørvoll et al., 2022). Thus, the body’s being in the world and sensorimotor life is regulated from the very beginning of life by movement experiences (Gallagher, 2005; Di Paolo et al., 2017). In children as evolving autonomous adaptive systems, movements and the registration of those movements foster the self-organizing development of neuronal structures significant for sensorimotor coordination, actions, as well as the way children become conscious of themselves, their interaction with others, and how they engage with the world through participatory sense-making (Gallagher, 2005; Quintero and De Jaegher, 2020). Movements are not pregiven capacities and are not primarily determined by previous experience. Rather, movements are something children experience and learn in various contexts by moving themselves as embodied agents (Sheets-Johnstone, 2010). For example, a typically developing child who explores herself and her surroundings through touch, vision, and varied and adaptive movements will discover new kinesthetic possibilities fostering body schema and body image. This enables the child to develop ongoing engagement with her environment, tasks, and others through participatory sense-making processes. Thus, variability in posture and movements is an essential element required to support motor control in typically developing children and drive adaptive changes for increasingly demanding tasks and participatory engagements as they relate to the child’s maturing nervous system, biomechanical properties of the growing musculoskeletal system, and environmental dispositions (Palisano et al., 2023). However, a child developing atypically, e.g., a child with CP who has impaired postural control and reduced muscle coordination, will show reduced variability and adaptability in her movement repertoire affecting how her body schema and body image evolve and the child will therefore experience fewer new kinesthetic opportunities and produce less varied movements (Hadders-Algra, 2018) making it challenging to co-constitute meaning with others in different contexts and environments.

The child as an embodied subject in its full sense is not just a matter of sensorimotor schema and body image but includes bodily affective states such as emotional factors, motivational dimensions, fatigue, pain, etc. Also, the perceptual sense of the possibility of executing movements and actions will affect the child’s engagement and participatory sense-making. Thus, interest, motivation, and affect are important bodily and emotional states that drive the child’s ability to understand and participate in interactions of concern. For example, a child with bilateral spastic CP may perceive a toy too far away and difficult to reach informing (pre-reflectively) her affective judgment not to reach for it. Hence, simply emphasizing the facilitation of the environment with toys to foster motor control in children may therefore not be sufficient.

The bodily states of emotions engender powerful agency and therefore support the child’s subjective desire to explore. The environment may afford many possibilities for exploration and action, but the child does not approach her environment merely based on inherent desire alone but also on what is achievable. Additionally, each possibility in the environment, whether an object or individual, will have its own value to the child. This will be based on what matters to the child as well as her interest in and/or the ability to interact with the object or person. By attributing properties of the world (objects, toys, other people, etc.) as being novel, difficult, complex, and/or unexplored, these properties are more likely to be of interest creating transformative changes in the embodied child as the coupling to her environment changes. Interest is therefore related to increased focus, modulated participatory sense-making, motivated engagement, and self-initiated actions. As children progress and transform their experiences, perceptions, and actions will shift and accordingly their goals and intentions start to operate in different ways. Further, individual affect (i.e., emotion, facial expression, posture, etc.) will differ from one person to another, between situations, relationships, contexts, and time of the day. Possibilities are therefore not equally affordable and will therefore affect body states that foster engagement and participatory sense-making (Colombetti, 2014).

In therapy then, according to the enactive approach the PT and the child are considered two interacting autonomous embodied agents enacting each other through various forms of participatory sense-making processes. The PT is part of the interaction in which individual sense-making will be affected by the activity of the other(s) to varying degrees (De Jaegher and Di Paolo, 2007). Sometimes the mutuality in the participatory sensemaking processes is high and regulatory while other times the mutuality plays out in a low influential way.

### 2.2. Movement is the change

Through movements children experience and discover the kinesthetic possibilities of their own bodies, coordinating and regulating themselves to others and/or to the environment. How a child moves determines what she perceives and vice versa. This is also a concept inherent in the dynamic systems approaches, but movements are understood differently in the dynamic systems approaches (as part of the objective body) compared to the enactive approach (the lived body). In the enactive context, movements are phenomenologically anchored in the lived body through pre-reflective self-perception and kinesthesia (Sheets-Johnstone, 2019). Each movement has a particular flow, e.g., when the child reaches for something, she forms and adapts her hand accordingly to what she is reaching out to grasp (round, square, heavy objects, etc.). Movements are in this manner closely related to proprioception and kinesthesia. Proprioception is the child’s emerging inner sense of the position of her body and limbs, while kinesthesia is the child’s emerging proprioceptive sense of her own movements (Gallagher, 2012). Through explorations of her own movements the child kinesthetically learns to *feel* her movements and proprioceptively learns to perceive the body postures which will optimize her movements. Thus, bodily movements involving proprioception and kinesthesia shape the child’s knowing and the process of knowing resides in her body and in her movements while she is interacting with her surroundings (Gallagher, 2005). Furthermore, cognition, the child’s learning or know-how about the world and herself, goes through the developmental progression of ‘I move’ – ‘I do’ – ‘I can’, also referred to as ‘thinking’ in movement (Sheets-Johnstone, 2019).

According to the enactive perspective, the ‘I’ in this context refers to the child as an embodied agent rather than simply mental consciousness (Gallagher, 2012). The child is pre-reflectively aware that she is moving, and this pre-reflective awareness is interwoven in experience itself. Let us use the example of a child, who has progressed from spontaneous arm movements to more targeted arm movements, for example grasping a toy. When the child initiates reaching by moving her arm (‘I move’) it enables her to ‘reach out’ for the toy placed in front of her body (‘I do’) and eventually, as she manages to grasp the toy and manipulate and cause the toy to move, her success provides a sensation of embodied knowledge that fosters agency (‘I can’) (Sheets-Johnstone, 2010, 2019).

Spontaneous movements, in general and how they are related to agency, play an essential part in initial learning throughout childhood. The ‘I move’ is therefore an important precondition for all learning, the foundation of ‘I can’ (Sheets-Johnstone, 2010, 2019). The ‘I do’ is related to movements (not actions or tasks) that have specific qualitative characteristics, e.g., whether the infant reaches quickly or slowly, succeeds, or fails. Through these movements, she will become kinesthetically sensitive to the properties, i.e., shape, weight, and hardness of the toy she grasps. As the infant continues to move, her kinesthetic repertoire will evolve into a vast range of movement dynamics, all of which will build kinesthetic memory over time providing her a sense of agency or ‘I can’ (Sheets-Johnstone, 2010, 2019).

The ‘I can’ is further defined as capabilities for movements that correlate with the state of the body and the affordances in the world (Sheets-Johnstone, 2010; Gallagher, 2012). In that context, the environment is not just a pregiven meaning waiting to be extracted by the child. Neither does the child passively receive information from her environment but rather actively participates in the generation of meaning in what matters to her and appears relevant and significant for her goals and intentions (Di Paolo et al., 2010). The softness, hardness, etc. of toys and other objects in the world are not to be found ‘in them’ but in how these objects respond to the child’s active manipulation (touching, probing, shaking, squeezing, etc.) through her perceptual bodily movements of ‘I can’ (Sheets-Johnstone, 2019; Sørvoll et al., 2022). However, it is not simply the size, shape, color, etc. of objects combined with the child’s ability to extend her arm that makes objects graspable. If the child for example has poor trunk control and cannot organize sufficient proximal stability for distal arm mobility (reach and grasp) (Shumway-Cook et al., 2023), then objects may not appear ‘graspable’ and thus of less value and interest. Hence, there will always be a mutual modulation between the child’s directedness (intentionality), her movements and how she qualitatively experiences what *she* is like as she moves, e.g., reaches for the toy (Gallagher, 2012). Hence, the ‘I can’ is a related aspect of the body schema which enables the child to explore her potentials and her surroundings through perceptions and bodily movements (Gallagher, 2005). Accordingly, the body finds answers to what it should do through movements - a kinesthetic experience, which allows the child to feel or perceive qualitatively through spatial and temporal dynamics (Sheets-Johnstone, 2010, 2019). Based on the motor control of the child, the movements may be efficient and effective or inefficient and potentially ineffective.

According to dynamic systems approaches (Palisano et al., 2023) bodily movements are commonly related to rhythm, tempo, length, synchronization etc., elements of structural importance for movements of the (living) biomechanical body. On the other hand, the enactive approach relates movements to temporality by referring to the phenomenological understanding of the lived body and how the child experiences order involved in temporal flow during movements, i.e., the child’s perceptions of opportunities in the present and future based on experiences from the past blend together as one experience (Gallagher, 2012). Movements consist of kinesthetic melodies (Sheets-Johnstone, 2019) that flow by in time and constitute their own temporal structures simultaneously just as the child’s experiences are constantly changing. The lived body knows or anticipates what is going to happen before movements occur. Regarding motor control the lived body needs to monitor previous movements by recognizing its trajectory toward its present state to anticipate future goals toward which the body is moving (Gallagher, 2012). While preconditions of the living and lived bodies enable and constrain the child’s agency in the present (here-and-now), perceived future opportunities motivate movements and actions.

To extend our understanding of the development of motor control and skill acquisition in children, we have in this and previous sections, described and discussed contributions of dynamic systems approaches and where they fall short incorporating the complex role of ‘the self’ in the development of motor control. By describing and explaining the enactive concepts of embodiment, autonomy, and participatory sensemaking, we hope we have shown how this theoretical framework can complement dynamic systems approaches and add a new dimension to the understanding of the role of the child in therapy. We have explained the relevance of the enactive approach as it relates to motor control and skill acquisition in children with both typical and atypical development. In the following section, we will apply the expanded insights drawn from the enactive approach to pediatric physical therapy.

## 3. The child’s embodied becoming and implications for physical therapy

Pediatric physical therapy is a relational and complex practice providing therapy to children of all ages and motor challenges (Sørvoll et al., 2022). Although these children with motor challenges have problems with functional skills, they also have bodily prerequisites. CP is the most common disability in children and constitutes a population of children that PTs encounter in their clinical practices. Children with CP have impairments in many areas, i.e., motor, social, communication, and cognitive which impact their way of perceiving and moving, and thus exploring their own bodies, movement possibilities, and their surroundings (Palisano et al., 2023). Therapy for these children is rooted in the framework of dynamic systems approaches, as is much of PT practice. However, from the viewpoint of the enactive approach we find two important under-considered aspects of the dynamic system approaches that hold significance for clinical practice: the lived body and situatedness of the child as well as the role of interactive factors.

By seriously considering the lived body and situatedness of the child in our therapeutic plans or interventions, the child becomes a co-creator of her own therapy. In this respect the child is involved in her own therapy as a bodily agent, and her presence is both embodied and enactive. The latter implies that the child shapes and actively contributes to her own development of motor control and skill acquisition by incorporating her own perspective of the significance of what matters to her and her need to self-sustain in any situation. It is an intricate connection between the child’s subjectivity and the interaction processes between the child and the PT (Øberg et al., 2013; Sørvoll et al., 2019). Situatedness involves both the child and the PT in an intersubjective bodily practice enabled and restricted by relational and contextual complexity through participatory sense-making (Øberg et al., 2013). Consequently, the PTs degree of involvement in the interaction with the child is a significant contributor for what evolves during therapy and fosters the child’s sense-making. In addition to what the child may verbally articulate, expressions of her lived body, such as gaze, facial expressions, gestures, postures, and bodily movements, are important parts of what the child communicates and contributes to the clinical encounter with the therapist and hence the therapist’s sense-making (Øberg et al., 2015; Sørvoll et al., 2022). However, children with neurological impairments often experience disturbances in perceptual and sensory networks and movements (Palisano et al., 2023) which may change their embodied intentionality. In this regard, intentionality is not reduced to pure mental consciousness but is a motor intentionality constituted through sensorimotor and affective processes (Gallagher, 2012). Although all children have an innate ability to participate in and communicate through bodily intersubjective processes, children with sensorimotor challenges often show subtle and less defined bodily expressions and less ability to regulate themselves (Blanchard and Øberg, 2015; Palisano et al., 2023). It is therefore particularly important that PTs acknowledge and take into consideration the children’s bodily expressions of their lived bodies.

Placing the child’s body in the center of the development of motor control and skill acquisition, encourages the PT to act in response to the child’s bodily expressions, movements, choices, needs, and motor challenges. This presumes the PT can arrange both the task and the environment in addition to perceive, anticipate, and guide the child (Sørvoll et al., 2019). Taken from this perspective therapy is thus broadened from the contemporary viewpoint of child-initiated problem-solving approaches without touch from a therapist, to include interventions that happen between child, PT, and environment as participatory sense-making processes in interactional-embodied practices. Interactional-embodied practices allow hands-on approaches to blend with established approaches (Sørvoll et al., 2022). The interaction processes between the child and the PT can then involve coordinated and adjusted movement loops like a ‘dance’ where they perceive and touch each other, while simultaneously being perceived and touched by the other (Sørvoll et al., 2022). In this ongoing ‘dance’, there are various, often unarticulated choices to be made by both therapist and child. We will present two hypothetic examples from pediatric physical therapy to illustrate how various degrees of mutuality and incorporeity have significance for what is created through participatory sensemaking processes.

**Figure 3:**
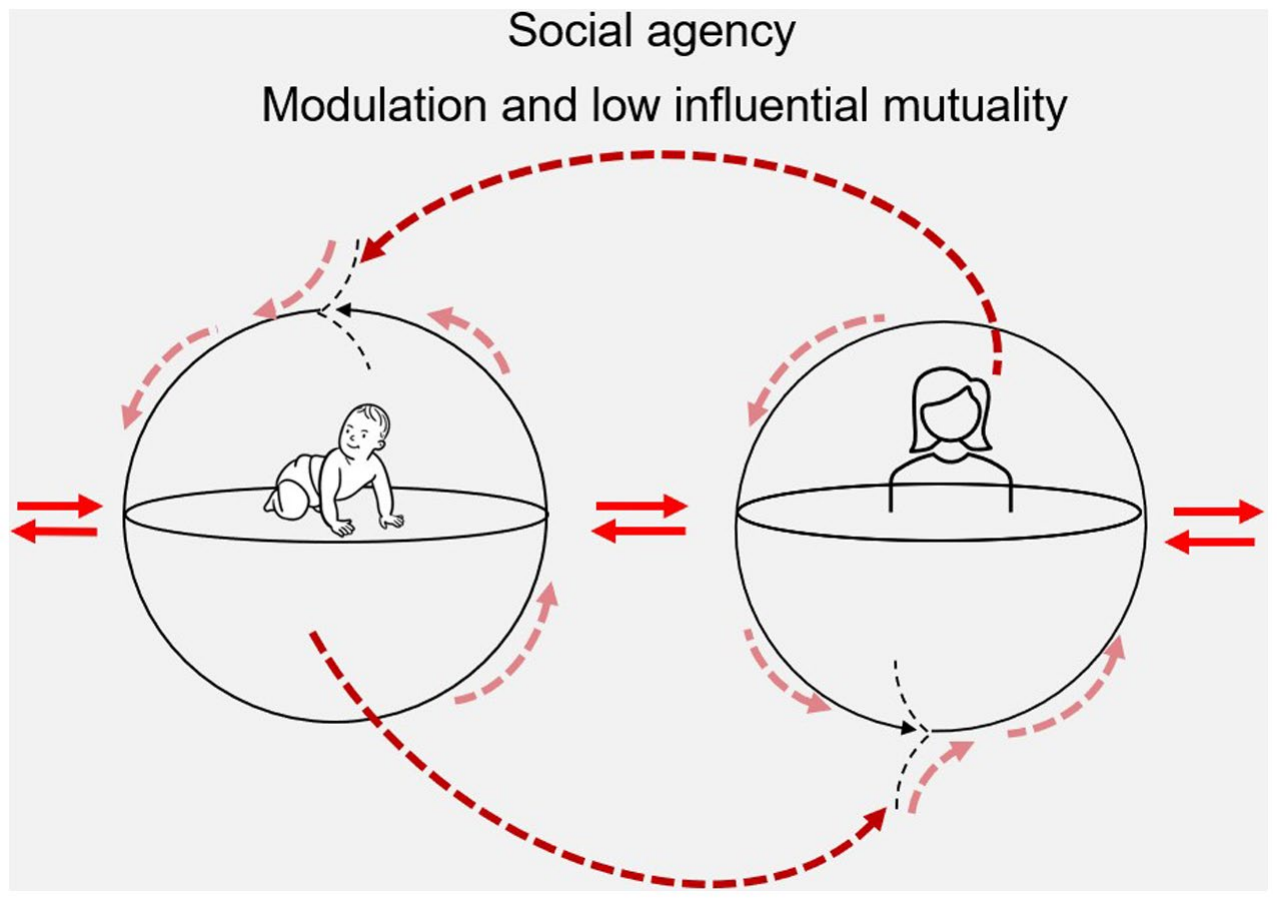
This figure is inspired by the work of Di Paolo et al. (2017) and shows two autonomous embodied agents (e.g., child and PT) enacting each other through simple influence. Sensorimotor coordination of movements and actions (dark red dashed arrow) in one of the agents (e.g., the PT) influences and enables sensorimotor coordination of movements and actions in the other agent (e.g., the child) (pink dashed arrows). Accordingly, sensorimotor coordination of movements and actions in the second agent (the child) influences the sensorimotor coordination of movements and actions in the first agent (the PT).

We begin with a situation characterized with low influential mutuality. Consider a PT who modifies the environment (the space) in a certain way (a mat to sit on and a small positioning pillow to prevent falling backwards) to enable a sitting child, who is delayed in her motor development, to achieve the task of reaching and gripping a toy placed on the right side slightly in front of her body. The long-sitting child (with her legs in line with her body) initiates a reaching movement with her right arm in a jerky, slow and stereotypical manner but fails due to reduced trunk control and inability to laterally weight shift in sitting. Consequently, the child’s trunk on the right side laterally flexes to the side in an effort to improve stability during reaching, causing her to fall to the right. The PT responds by replacing the child in the long-sitting position and places the toy on a small box closer to the child (still on her right side). Once more, the child initiates reaching movements in the same jerky, slow and stereotypical movement pattern previously used, but this time she succeeds in grasping the toy as the reaching distance, the height, and the location of the toy have been modified. The PT and child practice and repeat this activity over and over while the child continuous to move her arm in a stereotypical movement pattern with her trunk laterally flexed. This situation illustrates a PT who is not directly addressing the child’s lived body to regulate her interaction with the environment (toy) but facilitates the likelihood of success of grasping by re-arranging the specificity of the task; the child’s and the PT’s sense-making remain poorly synchronized individual activities (see Figure 3).

**Figure 4:**
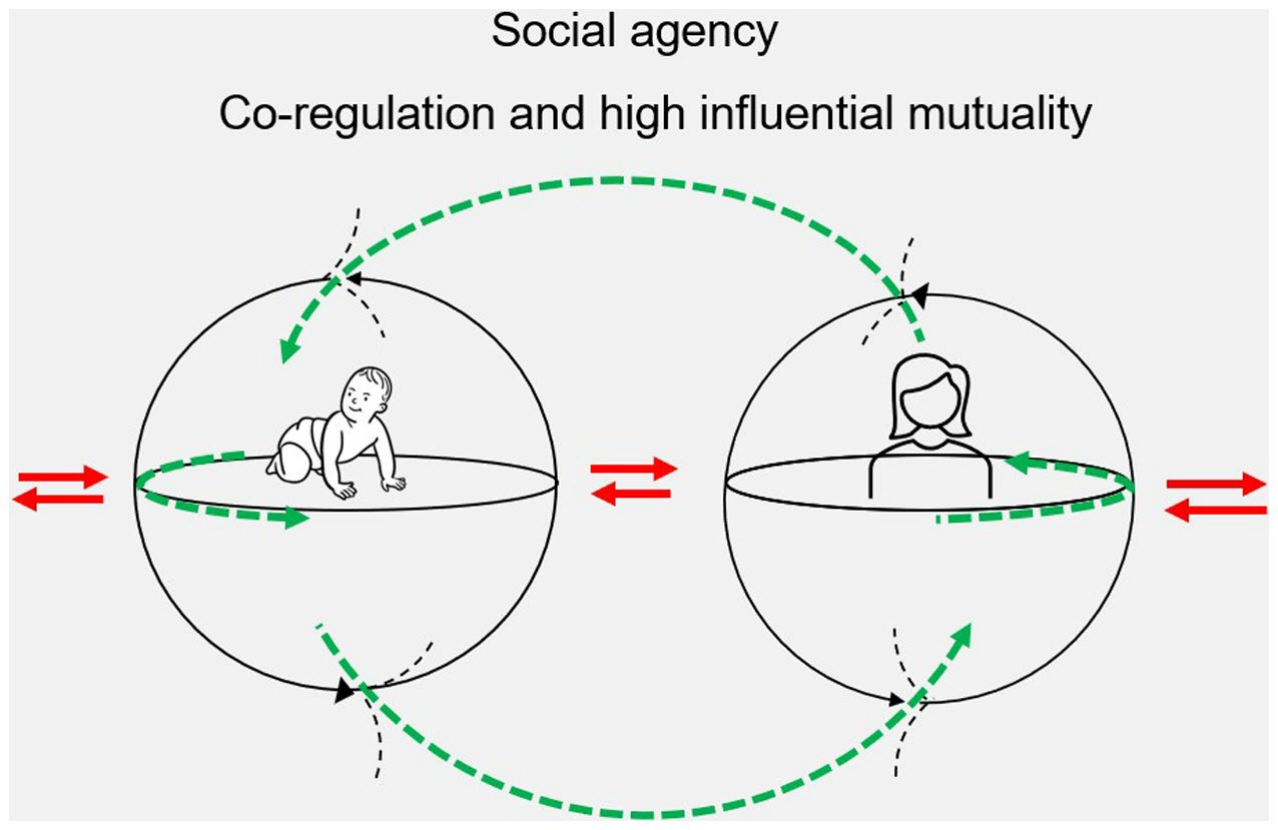
This figure is inspired by the work of Di Paolo et al. (2017) and shows two autonomous embodied agents (e.g., child and PT) enacting each other through specific co-regulation of perceptual movements. Sensorimotor coordination of movements and actions (green dashed arrow) in one of the agents (e.g., the PT) shapes and regulates the initiation of sensorimotor coordination of movements and actions in the other agent (e.g., the child) and vice versa.

Now, consider a situation where the starting point is the same as the situation above, but this is a scenario of high influential mutuality. The PT sits on the floor behind the long-sitting child. The PT places her hands lightly around the child’s trunk to provide sensory inputs to facilitate and guide trunk alignment improving postural stability and bodily awareness. The child can reach with greater fluidity and achieve the toy without falling sideways. In this context, facilitation and guidance should not be interpreted as a static support but rather an intervention to recruit motor activity and provide the child with a perceptual awareness of the optimal positioning to achieve the toy without falling. In this way, the child learns to reach while activating the postural mechanisms required to sustain her sitting posture. Based on the child’s bodily responses and what the PT comprehends through vision and touch and anticipates and perceives, the PT continuously adapts the position and compression of her hands as well as the position of the toy supporting and guiding until the child ‘takes over’. Over time, the PT reduces the control and pressure or even removes her hands, and the child is encouraged to experiment with her newly acquired kinesthetic experiences and developing trunk control as she reaches for the toy in different locations. This type of interaction further expands into a bodily interplay between the PT and the child where the PT alternates between being hands-on and hands-off while the child repeatedly reaches more smoothly and with greater speed and accuracy for the toy. This situation illustrates a child and a PT intervening in each other’s enactments through specific co-regulation of perceptual bodily movements; they are directly participating in the joint process of sense-making where the whole sense-making activity becomes a shared one (De Jaegher and Di Paolo, 2007) (see Figure 4).

The above two situations reflect how sense-making arises differently from two seemingly similar but contrasting intersubjective contexts, i.e., different child-environment relations generated different sense-making processes. In both cases the children embraced a perspective on the world by grasping the toy placed in front of them. However, the modulatory engagements in the interactions, i.e., the children’s moderation and incorporation of the sensorimotor flow from the environment into their movements, was different. Although both children achieved the end-goal of grasping the toy, the intent and structure of the therapy delivery placed different demands on their systems, giving rise to different circular coupling to the world and to sense-making.

In the first case, the PT only facilitated the task and the environment. Therefore, the role of the PT was to arrange the task and environment in relation to the child’s functional ability. The child struggled with her own body to complete the task. The adaptability and state of her living body were challenged resulting in precarious balance and slow labored movement. The child did not have the ability to vary her own movements and thus regulate her circular coupling to the environment. She was more dependent on the environment and task being regulated to her body to achieve the task. The child remained in her already established movement patterns with no improvement in her reaching movements. In that respect, the task-oriented approach fostered only the child’s kinesthetic feeling of ‘I move’ and ‘I do’ because the child was unable to adapt her own body to the task but rather depended on the task being adjusted to her body and her limited movement abilities. In other words, the child as an autonomous system was not addressed rendering her incapable to adaptively regulate her coupling with her environment.

In contrast, in the second example, the child was supported by the PT’s guided facilitation. Although the child was also vulnerable, she managed to modulate her coupling to the environment in a more adaptive manner. We suggest the PT’s facilitation provided sensory input to the child’s emerging body schemas enabling her to regulate her body and actively move her arm and trunk within previously unreachable ranges. The interplay between the child, the PT, and the task, constituted a performing system that enabled the child to explore the possibilities of her own body and to develop new kinesthetic experiences and her own agency. The mutuality in the situation was high and regulatory and contributed to the organization of the child as an autonomous adaptive system. Thus, the child’s embodied intentionality changed allowing her to interact more directly with her surroundings. This fostered promising, immerging, or nascent changes in the child’s ability to reach and grasp., which over time can develop in self-sustaining patterns or habits that will constitute an emerging network of body schemas that can be applied to similar activities (Di Paolo et al., 2017). Additionally, these movement improvements can also be understood as fostering the child’s kinesthetic feeling of ‘I can’ and thus her sensorimotor agency through optimal movement strategies.

To reinforce the significance of the use of (pre-)reflective, guided facilitation, there are several studies which substantiate the importance of various forms of sensory trunk support in children developing typically and atypically (Rachwani et al., 2015; Santamaria et al., 2016; Shumway-Cook et al., 2023). For example, a study, with typically developing children observed how parents provided support through various forms of physical touch and handling during their first year of life. Parents handling was strongly correlated with a standardized trunk assessment and showed that parents intuitively anticipated and provided support and hands-on adjustments consistent with the child’s increasing postural control (Duncan et al., 2018). Having this knowledge about typical motor development in children, it is a paradox that PTs working with children with neurological impairments such as CP, should be discouraged from using hands-on approaches, which are consistent with handling behaviors parents intuitively demonstrate with typically developing children.

For the processes of embodied interactions during clinical encounters, the PT’s embodied knowledge becomes central to support and guide the application of her clinical skills with the goal of co-creating optimal changes in the child’s motor function and participation. Additionally, during clinical encounters, the PT’s embodied knowledge should also be directed toward the incorporation of activity and skills to support the child’s state, organization, and agency. Consequently, in embodied interactions with the child the PTs knowledge will rise above a purely cognitive activity to an embodied ‘dance’ with the child and her bodily expressions and intentional movements. In this respect, interventions are not about knowing and performing methods but the PT’s perception, understanding and anticipation of the child’s bodily expressions and motor intentions.

## 4. Final remarks

The enactive approach combines epistemological and ontological concerns of sensorimotor life, allowing us to conceive children as autonomous, adaptive embodied agents by viewing the relationship between the child’s mind–body and the world as dynamic, temporal, and co-evolving. In that respect, the enactive approach offers PTs and other health professionals integrated insights from various theoretical fields, as they relate to the development of motor control and skill acquisition. The enactive approach offers an alternative perspective to guide and comprehend clinical practice from the perspective of the child through participatory sensemaking. Our aim was not to highlight one theoretical position over another but rather to present the common principles of contemporary frameworks of motor control and to expand them by incorporating an enactive understanding of the child as an embodied agent in the world. We believe that our recommendation of incorporating an enactive embodied view to therapy offers an innovative approach to practice and provides PTs and other health professionals the opportunity to challenge established ways of thinking and practicing. Finally, we would make clear that these embodied concepts should be understood as ongoing processes and can be integrated into clinical encounters with patients of any age.

## Data availability statement

The original contributions presented in the study are included in the article/supplementary material, further inquiries can be directed to the corresponding author.

## Author contributions

MS and GKØ were responsible for the conceptualization of the manuscript. Additionally, MS drafted and prepared the figures. All authors contributed to the preparation of the first draft, to the review and revision of the manuscript, approved the submission, and are accountable for all aspects of the work.
